# Minimally Invasive Endoscopic Approach to the Cervicothoracic Junction for Vertebral Osteomyelitis

**DOI:** 10.1155/2017/2495041

**Published:** 2017-12-11

**Authors:** Tadatsugu Morimoto, Masatsugu Tsukamoto, Tomohito Yoshihara, Motoki Sonohata, Masaaki Mawatari

**Affiliations:** Department of Orthopaedic Surgery, Faculty of Medicine, Saga University, 5-1-1 Nabeshima, Saga 849-8501, Japan

## Abstract

The selection of an anterior, lateral, or posterior approach to the cervicothoracic junction for surgical treatment of vertebral osteomyelitis is still a matter of debate. These ordinary approaches generally require an extensile exposure. This article describes a less invasive approach case of a vertebral osteomyelitis of T2/3 using a video-assisted operating technique of thoracic surgery (VATS). A 78-year-old female underwent anterior debridement and interbody fusion with bone graft at T2/3 using a lateral surgical approach through a right thoracotomy with VATS. The VATS through two small skin incisions in the axillary region provides a good view without requiring elevation of the scapula with extensile muscle dissection and rib resection. There was no complication without partial lobectomy due to pleural adhesion during the perioperative period. Currently, at 1 year after operation, the patient has no back pain with neurologically normal findings and no inflammation findings (CRP was 0.01 mg/dl). Although the operating field of the upper thoracic level in the lateral approach is generally deep and narrow, the VATS provides a good view and allows us to perform adequate debridement and bone fusion at the T2/3 level with a less invasive approach than those previously described anterior or laterally or posterior approach.

## 1. Introduction

Difficulties with exposure of the cervicothoracic junction (CTJ) are well known [1–9]. The selection of anterior, lateral, or posterior approach to the CTJ for surgical treatment of vertebral osteomyelitis (VO) is still a matter of debate [1–9]. These ordinary approaches generally require an extensive exposure, which can lead to significant morbidity [6–9].

Although the video-assisted operating technique of thoracic surgery (VATS) has become commonly used for middle or lower thoracic spine surgery [4, 5, 10, 11], few cases of VATS for upper thoracic spine surgery have been reported [1, 4]. In the present case, VATS was used for surgery at the CTJ for VO of T2/3 to minimize the approach and optimize visualization.

## 2. Case

**Figure 1:**
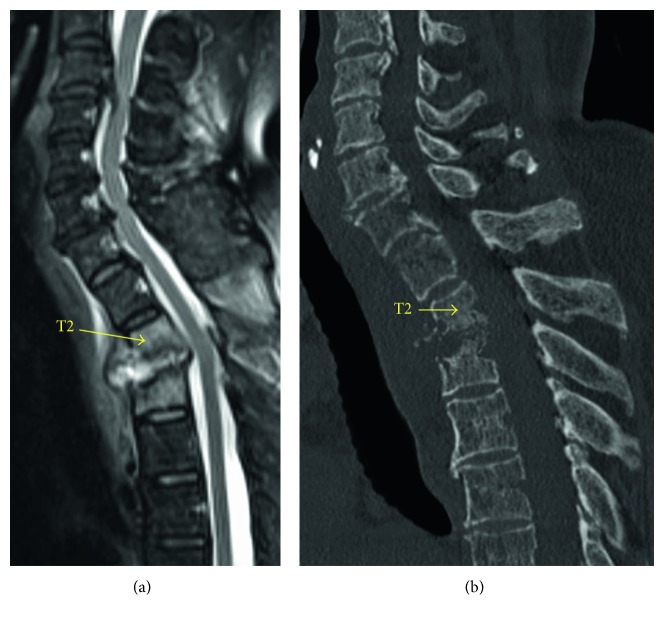
(a) Preoperative sagittal T2-weighted magnetic resonance image of the cervicothoracic spine shows vertebral osteomyelitis and intervertebral disk abscess of T2/3. (b) Preoperative sagittal CT shows bone destructions of the endplates of T2/3.

A-78-year-old woman with VO at L4-5 underwent laminectomy, debridement, and interbody fusion. Three months after the operation, she complained of persistent fever and back pain with no neurological deficit. Her C-reactive protein (CRP) was 18 mg/dl. Magnetic resonance imaging (MRI) showed no abscess in the lumbar spine, but it showed an abscess of T2/3 (Figure 1). CT demonstrated bone destructions of the endplates of T2/3 (Figure 1).

**Figure 2:**
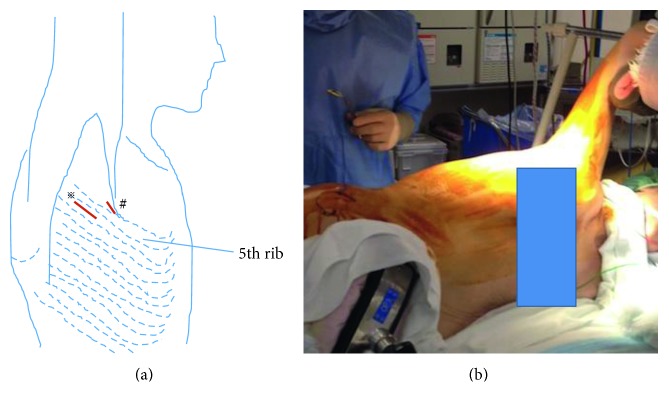
Schema of position and skin incisions. (a) At surgery, the patient's arm is positioned at 80° of abduction and neutral flexion, extension, and rotation due to make a 4 cm long skin incision as a utility port along the mid-axillary line between the fifth and sixth costal spaces in the axillary region (※), which was directly located on the T5/6 disc level. One additional incision was made along the anterioraxillary line between the fourth and fifth costal spaces in the axillary region (#). (b) Left lateral decubitus position.

**Figure 3:**
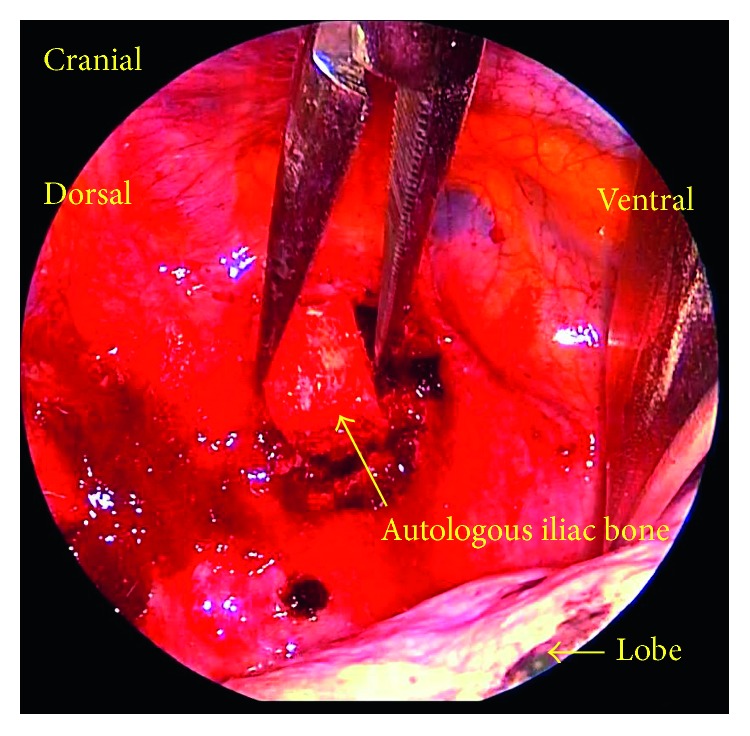
Anterior debridement and interbody fusion at T2/3 were performed using a video-assisted operating technique.

**Figure 4:**
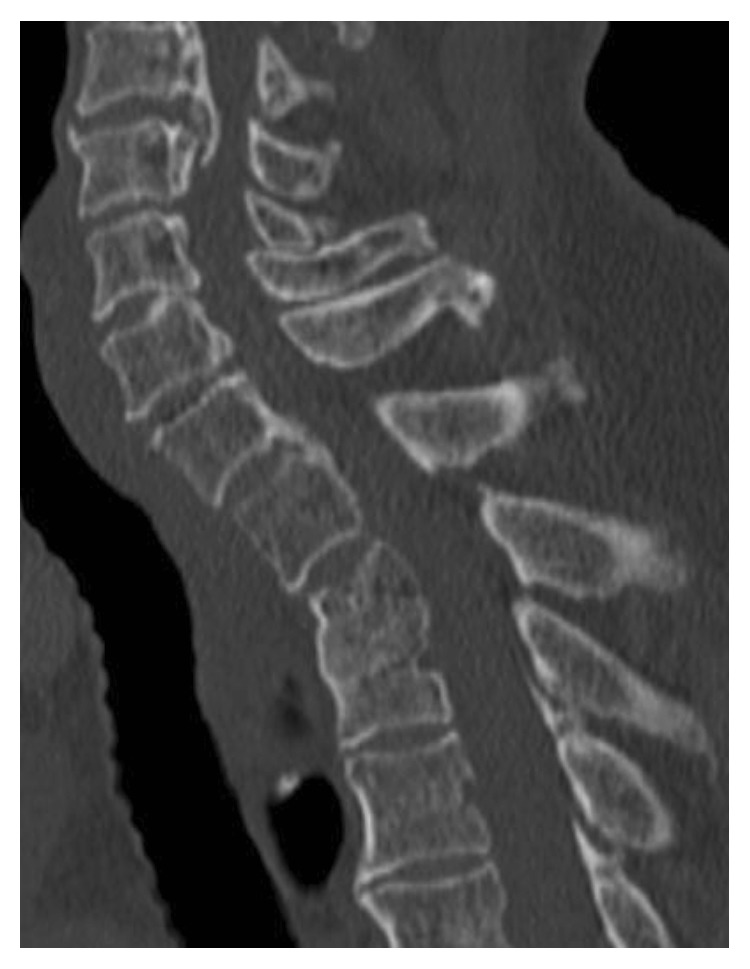
Sagittal CT of the cervicothoracic spine six months after operation shows bony fusion at T2/3.

Anterior debridement and interbody fusion at T2/3 were performed using a lateral VATS approach with left lateral decubitus position (Figures 2 and 2). Although the camera port was situated at the T4/5 level, the use of an endoscope allowed an extensive view. Thoracoscopic dissection for pleural adhesion and partial lobectomy at the T2/3 level were performed by a thoracic surgeon. The pus and visible necrotic tissues (nucleus pulposus and endplate cartilage) were scraped, and the intervertebral space was thoroughly rinsed with povidone-iodine saline. An autologous iliac strut and chipped bone were implanted into the evacuated space (Figure 3). The operating field was deep, narrow, and unfamiliar, which produced disorientation. Thus, several confirmations of the surgical anatomy with fluoroscopy were needed. The operating time was 151 minutes, and the blood loss was 120 g. There were no complications related to the partial lobectomy during the perioperative period. Pain was significantly reduced after operation. *Streptococcus anginosus* was identified. She was treated with 500 mg/day levofloxacin intravenously for 4 weeks followed by oral administration. At 1 year after the thoracic surgery, the patient had no back pain and no abnormal neurological findings. Her CRP was 0.01 mg/dl. CT showed bony fusion at T2/3 (Figure 4).

## 3. Discussion

Traditionally, the anterior approach to the CTJ has been preferred, but exposure is very difficult because of the many vital structures [2, 3, 6, 7]. Most of the previously described techniques are extensive and require osteotomy of the clavicle or sternotomy [6, 7]. Another risk of the anterior approach for VO at the upper thoracic may be leading to descending necrotizing mediastinitis, which can cause a life-threatening condition with high mortality [12]. Therefore, the anterior approach has been considered the last resort. The ordinary lateral approach requires elevation of the scapula with extensive muscle dissection and rib resection [8]. Although the posterior approach has gained popularity, it needs extended posterior instrumentation to cover multiple levels above and below the level of pathology [9]. Thus, all these ordinary approaches are difficult and potentially dangerous, which can lead to significant morbidity [6–9]. In the present case, a VATS approach to the CTJ that is less invasive than the previous approaches was used.

There are several advantages and disadvantages with VATS at the upper thoracic level.

First, although the operating field at the upper thoracic level in the lateral approach is generally deep and narrow, VATS allows excellent visualizations by using a 30° scope. However, the portals for the retractor and the suction instrument were situated caudally from the pathology level, and a simple surgery such as sympathectomy [13], herniotomy [5], and debridement for disk infection is possible, but T1, T2, or T3 corpectomy may be very difficult to operate from a low angle view.

Second, the VATS approach through small skin incisions in the axillary region, which naturally improve cosmesis, did not require elevation of the scapula with extensive muscle dissection and rib resection.

Third, compared to the posterior approach including posterior stabilization using percutaneous pedicle screws, the advantages of this operation were (1) direct access to the lesion, (2) no damage and thus preventing the spread of infection to the posterior structures, and (3) shorter segment stabilization [1–3], as only one-level fusion at T2/3 was needed in the present case.

Finally, spine surgeons should carefully coordinate with thoracic surgeons for safety because it can be more difficult to handle pleural adhesions due to chest surgery, trauma, or previous infection, as in the present case.

## 4. Conclusion

VATS is the key to providing a good view and to allowing adequate debridement and bone fusion at the T2/3 level with a less invasive approach than those previously described approaches.
